# Serum HE4 and CA125 combined to predict and monitor recurrence of type II endometrial carcinoma

**DOI:** 10.1038/s41598-021-01263-w

**Published:** 2021-11-04

**Authors:** Quan Quan, Qianqian Liao, Wanchun Yin, Shuwei Zhou, Sainan Gong, Xiaoling Mu

**Affiliations:** 1 Department of Gynecology, The First Affiliated Hospital of Chongqing Medical University, No. 1 Youyi Road, Yuanjiagang, Yuzhong District, Chongqing, 400016 China; 2 Department of Obstetrics and Gynecology, Chongqing Health Center for Women and Children, Chongqing, China; 3 Department of Gynecology, The First People’s Hospital of Chongqing Liangjiang New Area, Chongqing, China; 4 Department of Obstetrics, Chongqing Health Center for Women and Children, Chongqing, China

**Keywords:** Gynaecological cancer, Tumour biomarkers, Tumour heterogeneity

## Abstract

There is no recognized serum biomarker to predict the recurrence of endometrial carcinoma (EC). We aimed to explore serum human epididymis protein 4 (HE4) and cancer antigen 125 (CA125) as the biomarkers to predict and monitor recurrence of type II EC. 191 patients diagnosed with type II EC were involved for this retrospective study. Comparing recurrent with non-recurrent patients, HE4 levels resulted a statistically significant difference at primary diagnosis and recurrence, respectively (P = 0.002 and P = < 0.001), while CA125 levels resulted statistically significant (P = < 0.001) at recurrence. According to receiver operating characteristic curve analysis, the areas under the curve were significant for HE4 levels at primary diagnosis and recurrence predicting recurrence. Furthermore, CA125 levels at recurrence were significant. And the combination of both markers showed the higher sensitivity and specificity than single one. Patients with higher HE4 levels were associated with worse disease-free survival and overall survival, the opposite was true for patients with lower HE4 levels. The preoperative HE4 levels could be used to evaluate the risk factors of type II EC. Which suggested that HE4 levels might associated with the prognosis of type II EC. And combination of HE4 and CA125 could be applied to monitor recurrence during follow-up.

## Introduction

Endometrial carcinoma (EC) is the most common malignancy of the female genital tract and the fourth commonest cancer in women^1^, most of the EC diagnosed at the early stage, having a favorable prognosis with a 5-year overall survival of 74–91%^2^. However, 13–17% of the EC patients recur within the first 3 years^3,4^. EC is usually classified into two types: type I include grade 1 and grade 2 endometrioid carcinoma, and type II refers to grade 3 endometrioid carcinoma and special types (such as serous carcinoma, clear cell carcinoma and carcinosarcoma). Type I EC presents a good prognosis, and type II EC is susceptible to distant metastasis, recurrence and poor prognosis^5,6^.

Traditionally, EC patients followed up by imaging techniques, such as computed tomography (CT), magnetic resonance imaging (MRI) and positron emission tomography (PET)^7^. Yet, these approaches were high expenditure and not handy. Up to now, no consensus has been reached on the serological markers of EC. Overexpression in human epididymis protein 4 (HE4) and cancer antigen 125 (CA125) genes were found in EC patients through The Cancer Genome Atlas (TCGA) gene database (https://cancergenome.nih.gov/) and we found the significantly correlated with the overall survival of EC patients, which indicated that HE4 and CA125 were closely correlated with EC. HE4 and CA125 are commonly used in clinical practice. In clinical practice, HE4 and CA125 have been routinely used to diagnose many malignant tumors, especially ovarian cancer^8^. Despite previous reports, that the serum tumor markers CA125 and HE4, are significantly correlated with recurrence of EC^9,10^. Yet CA125 is easily affected by inflammation, endometriosis, and pregnancy, but HE4 is not^11^. In our knowledge, there is little study on the application of serum HE4 in type II EC.

HE4 is a part of the WAP four-disulfide core family of proteins, belongs to the protease inhibitors. It’s a secreted protein that is overexpressed in malignant tumors, especially in ovarian cancer and EC^12^.

Current guidelines of the National Comprehensive Cancer Network (NCCN) point out, Patients with EC after initial treatment, who experience symptoms such as abnormal bleeding again or imaging suggests recurrence, commonly, tend to distant metastasis however, leading to poor results of treatment, in the literatures^13^, the early detection and comprehensive individual treatment of local recurrence, will guide a better prognosis.

The aim of this study was to appraise the appropriateness of HE4 as an indicator for the follow-up of type II EC recurrence, and to evaluate the value of HE4 combined with CA125 in monitoring the recurrence of type II EC, to assess the optimal cut-off value of CA125 and HE4 for type II EC recurrence.

## Materials and methods

### Patients

191 patients with type II EC (median age 60 years, range 35–89 years), all accepted surgery treatment at the Department of Gynecology, The First Affiliated Hospital of Chongqing Medical University, between May 2011 and August 2019 were included in this retrospective analysis. The stage of the patients was assessed according to the International Federation of Gynecology and Obstetrics (FIGO) staging system^14^. Patients with progressive disease were excluded from the study. All of the patients accepted surgery at least included total hysterectomy and bilateral salpingo-oophorectomy, advanced-staged patients were also treated for cytoreductive procedures. Most of the patients accepted adjuvant treatment (chemotherapy and/or radiotherapy). Patients Follow-up implemented was from primary diagnosis until the last registered visit or death. Interval time was about 3–6 months for the first 3 years and twice or once a year till 5 years. The study was approved by the Ethics Committee of the First Affiliated Hospital of Chongqing Medical University and was performed in accordance with the tenets of the Declaration of Helsinki, and informed consents were obtained from all patients. Clinical information was collected from the Electronic Medical Record System of The First Affiliated Hospital of Chongqing Medical University.

### Quantitative determination of HE4 and CA125 in human serum

3 mL serum specimen was collected before surgery or during follow-up from each patient to detect serum HE4 and CA125 levels by chemiluminescence approach (Abbott Laboratories, US) on the fully automated Architect instrument. Sampling for patients at 3–6 month intervals for first 3 years followed by every 6–12 months thereafter during follow-up. The normal reference value was as follows: pre-menopausal HE4 ≤ 70 pmol/L, post-menopausal HE4 ≤ 140 pmol/L. CA125 < 35U/mL.

### Statistical analysis

All statistical calculations were performed with SPSS software (version 25.0; IBM SPSS). A probability of 0.05 was considered to indicate statistical significance. We used the Wilcoxon signed-rank test to estimate the association between serum HE4, CA125 and recurrence. We used receiver operator curves (ROC) were to evaluate the ability of serum biomarker to identify patients with recurrent disease. The optimal cut-off value of serum HE4 and CA125 levels were determined using the maximum Youden index (YI = Sensitivity + Specificity − 1). For survival analysis, disease-free survival (DFS) was defined as the time interval between the date of surgery and the date of recurrence or death/last follow-up, while overall survival (OS) was defined as the time interval between the date of diagnosis and the date of death or last follow-up. Survival models were fitted using the Cox proportional hazard models, while survival curves were drawn based on the Kaplan–Meier methods. All the models assumptions were tested.

## Results

A total of 232 patients were diagnosed as type II EC between 2011 and 2019, but 30 patients were lost at follow-up, 11 patients did not undergo surgery only chemotherapy treatment. Consequently, only 191 patients were involved in this retrospective study, Median follow-up was 27 months (range 2–94 months). 42 patients developed recurrent disease, and 42 patients died of the disease. The characteristics of patients are demonstrated in Table 1.

**Table 1:** Patients’ characteristics.

Age (years; median)	60 (35–89)
Variable	Data (n = 191)
Postmenopausal	147 (76.96)
**Histology**	
Endometrioid carcinoma grade 3	70 (36.65)
Serous carcinoma	78 (40.84)
Clear cell carcinoma	32 (16.75)
Carcinosarcoma	9 (4.71)
Neuroendocrine	2 (1.05)
**Myometrial invasion**	
Superficial	108 (56.54)
Deep	83 (43.46)
**Lymph node metastasis**	
Absent	138 (72.25)
Present	28 (14.66)
Unknown	25 (13.09)
**FIGO stage**	
I	91 (47.64)
II	26 (13.61)
III	63 (32.98)
IV	11 (5.76)

Numbers in parentheses are percentages.

Table 2 shows the correlation between serological markers and pathological factors of type II EC in each group. According to Wilcoxon test, serum CA125 and HE4 are significantly related to clinical pathological risk factors at primary diagnosis, such as depth of myometrial invasion, lymph node status and FIGO stage. The levels of serum HE4 and CA125 elevated significantly in the high-risk groups. In the process of analysis, we found a statistically significant difference between HE4 at initial diagnosis and at recurrence (P = 0.002, P = < 0.001), respectively, comparing recurrent and non-recurrent patients. Otherwise we found a statistically significant difference between CA125 at recurrence (P = < 0.001) comparing recurrent and non-recurrent patients.

**Table 2:** Pathological factors in relation to serum levels of HE4 and CA125.

Variable	CA125 (U/mL)	HE4 (pmol/L)	CA125 (U/mL)	HE4 (pmol/L)
	At primary diagnosis	At primary diagnosis	At recurrence	At recurrence
**Myometrial invasion**				
Superficial	22 (15–36)*	61 (44–88)*	13 (10–24)*	54 (37–69)*
Deep	32 (15–70)**	82 (53–126)**	14 (7–37)*	51 (41–93)*
**Lymph node metastasis**				
Absent	20 (14–33)*	64 (46–90)*	13 (8–23)*	52 (39–69)*
Present	43 (32–106)**	65 (49–179)**	14 (9–49)*	52 (38–79)*
**FIGO**				
I–II	19 (12–31)*	63 (46–88)*	12 (9–21)*	50 (39–66)*
III–IV	35 (24–95)**	71 (49–169)**	17 (9–50)**	53 (39–90)**
**Recurrence**				
No	25 (14–38)*	63 (46–89)*	12 (9–20)*	50 (38–60)*
Yes	30 (19–106)*	91 (55–162)**	100 (15–460)**	120 (57–510)**

Numbers are the median with the interquartile range (IQR).

Different superscript symbols (*,**) indicate significant differences between two groups (P < 0.05), either superscript symbols are same mean the difference is not significant (P > 0.05).

**Figure 1:**
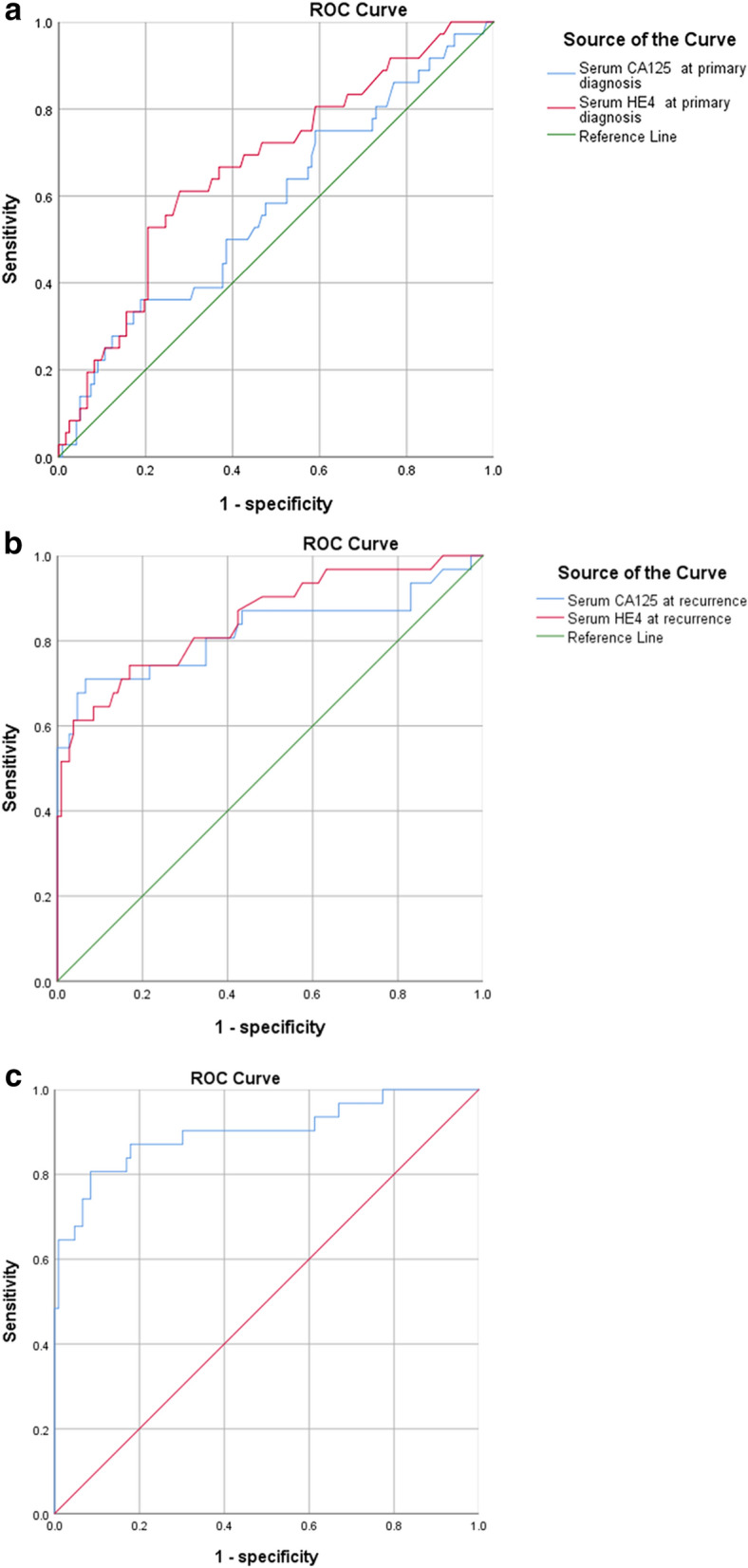
The ROC curve of serum CA125 and HE4 at primary diagnosis (**a**), CA125 and HE4 at recurrence (**b**), the combination of CA125 and HE4 at recurrence (**c**).

At primary diagnosis time point, in our study, HE4 levels above 89.50 pmol/L demonstrated sensitivity of 61.1% and a specificity of 72.1% in predicting EC recurrence. Furthermore, we found that a HE4 cut-off value of 92.50 pmol/L at recurrence time point is able to monitor patients at recurrence or not, with a sensitivity of 61.3% and a specificity of 96.2%, and a CA125 cut-off value of 32.45 U/mL, with a sensitivity of 71.0% and a specificity of 93.4%. The areas under the curve (AUCs) were not significant for CA125 at primary diagnosis. The combination of both biomarkers was higher associated with recurrence, with a sensitivity of 80.6% and a specificity of 91.5% (Table 3 and Fig. 1).

**Table 3:** Prediction performance of type II EC recurrence assessment by serum CA125 and HE4.

	Cut-off value	Sensitivity (%)	Specificity (%)	AUC (95% CI)	P value
CA125 (U/mL) at primary diagnosis	46.50	36.1	81.1	0.578 (0.469, 0.686)	0.158
HE4 (pmol/L) at primary diagnosis	89.50	61.1	72.1	0.669 (0.569, 0.769)	0.002
CA125 (U/mL) at recurrence	32.45	71.0	93.4	0.822 (0.714, 0.930)	< 0.001
HE4 (pmol/L) at recurrence	92.50	61.3	96.2	0.850 (0.765, 0.935)	< 0.001
Both CA125 and HE4 at recurrence		80.6	91.5	0.900 (0.825, 0.974)	< 0.001

*CI* confidence interval.

**Figure 2:**
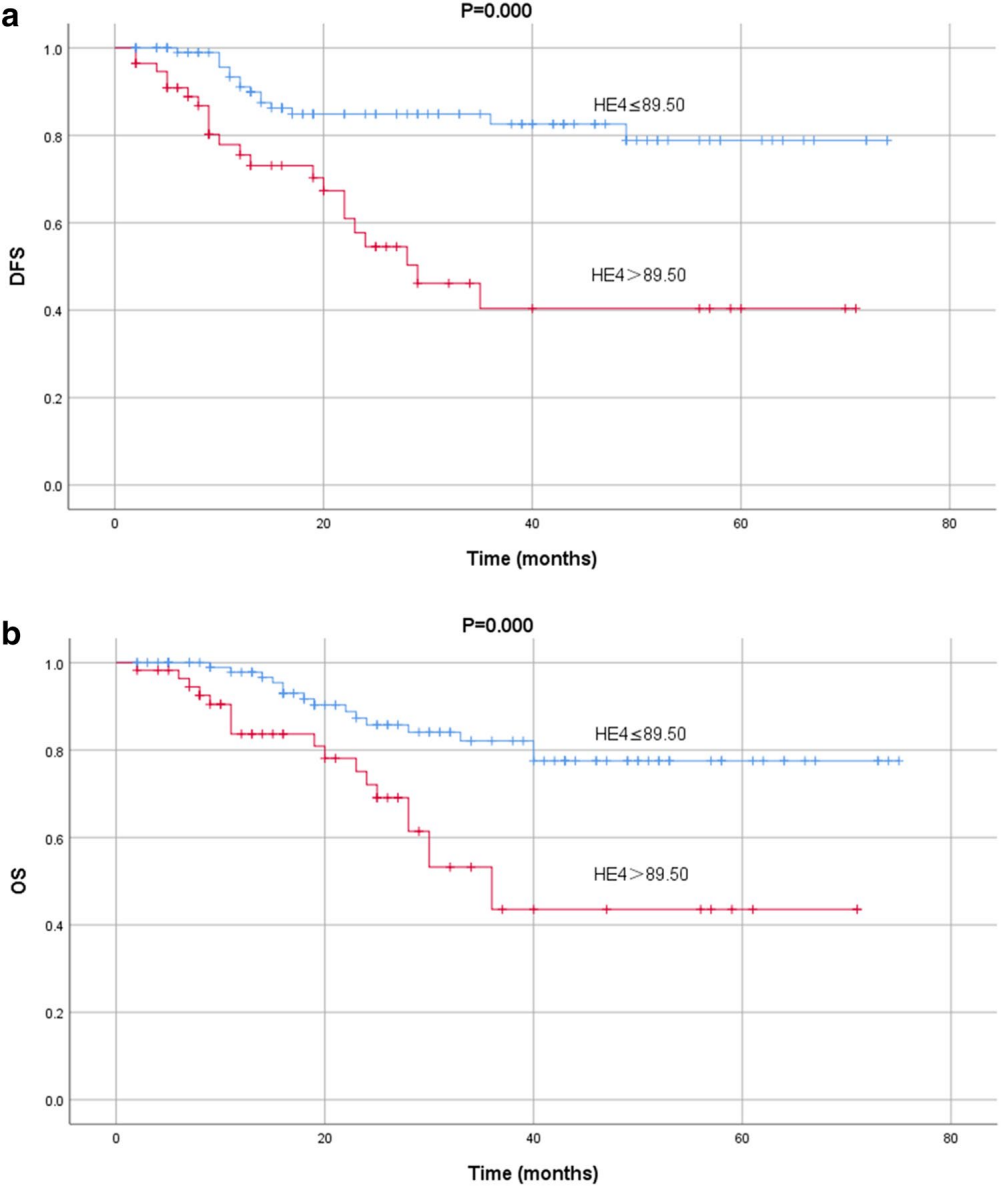
Kaplan–Meier curves for the disease-free survival (**a**) and overall survival (**b**), according to HE4 at primary diagnosis. Cut-off values were 89.50 pmol/L.

According to the cut-off level for preoperative serum HE4, we found that patients with higher HE4 levels were associated with worse DFS and OS, while patients with lower HE4 levels were associated with better DFS and OS. (Fig. 2) The univariate cox analysis was used to analyze the clinicopathological factors may affect the prognosis of EC. (Table 4) In addition, we found that the serum HE4 levels exceed 92.50 pmol/L, CA125 levels greater than 32.45 U/mL at recurrence were independent factor for DFS and OS (Table 5).

**Table 4:** Univariate prognostic analyses.

Parameters	HR	DFS 95% CI	P	HR	OS 95% CI	P
**Age**						
≤ 60 years	1			1		
> 60 years	2.702	1.404–5.200	0.003	2.056	1.093–3.868	0.025
**Histology**			0.009			0.039
Endometrioid carcinoma grade 3	1			1		
Serous carcinoma	2.533	1.177–5.453	0.017	1.286	0.644–2.567	0.476
Clear cell carcinoma	0.862	0.265–2.801	0.805	0.536	0.176–1.629	0.272
Carcinosarcoma	4.895	1.505–15.921	0.008	2.675	0.879–8.136	0.083
Neuroendocrine	8.488	1.058–68.131	0.044	10.318	1.304–81.642	0.027
**Myometrial invasion**						
Superficial	1			1		
Deep	2.640	1.414–4.929	0.002	2.378	1.275–4.437	0.006
**Lymph node metastasis**						
Absent	1			1		
Present	2.833	1.277–6.284	0.010	1.957	0.833–4.599	0.124
**FIGO stage**			< 0.001			< 0.001
I	1			1		
II	2.108	0.633–7.016	0.224	1.633	0.518–5.141	0.402
III	7.085	3.216–15.609	< 0.001	4.050	1.966–8.345	< 0.001
IV	3.930	0.828–18.662	0.085	7.005	2.194–22.361	0.001
**CA125 (U/mL) at primary diagnosis**						
≤ 46.50	1			1		
> 46.50	3.444	1.804–6.573	< 0.001	2.776	1.420–5.427	0.003
**HE4 (pmol/L) at primary diagnosis**						
≤ 89.50	1			1		
> 89.50	3.749	1.937–7.259	< 0.001	3.176	1.607–6.279	0.001
**CA125 (U/mL) at recurrence**						
≤ 32.45	1			1		
> 32.45	15.507	7.112–33.814	< 0.001	8.059	3.922–16.563	< 0.001
**HE4 (pmol/L) at recurrence**						
≤ 92.50	1			1		
> 92.50	12.317	5.988–25.337	< 0.001	7.042	3.395–14.607	< 0.001

*HR* hazard ratio, *CI* confidence interval.

**Table 5:** Multivariate prognostic analyses.

Parameters	HR	DFS 95% CI	P	HR	OS 95% CI	P
**Age**						
≤ 60 years	1			1		
> 60 years	0.170	0.023–1.232	0.079	0.523	0.116–2.349	0.398
**Histology**			0.576			0.082
Endometrioid carcinoma grade 3	1			1		
Serous carcinoma	0.899	0.250–3.236	0.870	0.304	0.073–1.264	0.101
Clear cell carcinoma	0.679	0.124–3.732	0.656	0.161	0.026–1.006	0.051
Carcinosarcoma	0.429	0.064–2.868	0.383	0.292	0.046–1.876	0.195
Neuroendocrine	17.720	0.398–788.824	0.138	5.201	0.190–142.246	0.329
**Myometrial invasion**						
Superficial	1			1		
Deep	4.460	0.990–20.095	0.052	2.138	0.534–8.558	0.283
**Lymph node metastasis**						
Absent	1			1		
Present	0.310	0.063–1.540	0.152	0.082	0.014–0.473	0.005
**FIGO stage**			0.562			0.014
I	1			1		
II	0.362	0.059–2.206	0.270	0.790	0.105–5.912	0.818
III	1.126	0.193–6.575	0.895	12.443	1.782–86.877	0.011
IV	2.371	0.187–29.994	0.505	24.520	2.625–229.027	0.005
**CA125 (U/mL) at primary diagnosis**						
≤ 46.50	1			1		
> 46.50	2.632	0.666–10.403	0.167	1.021	0.235–4.432	0.978
**HE4 (pmol/L) at primary diagnosis**						
≤ 89.50	1			1		
> 89.50	1.400	0.356–5.499	0.630	3.001	0.651–13.837	0.159
**CA125 (U/mL) at recurrence**						
≤ 32.45	1			1		
> 32.45	7.864	2.460–25.144	0.001	8.835	2.521–30.969	0.001
**HE4 (pmol/L) at recurrence**						
≤ 92.50	1			1		
> 92.50	15.565	2.491–97.251	0.003	4.493	1.047–19.289	0.043

The total number of events were both 21 for recurrence and death.

*HR* hazard ratio, *CI* confidence interval.

## Discussion

In our study, we found that the preoperative serum HE4 levels of patients without recurrence were significantly lower than those with recurrence. The higher the preoperative serum HE4 levels, the worse the DFS and OS, which suggested that the preoperative serum HE4 levels could be applied to predict the prognosis of patients. There was no statistically significant difference in their preoperative serum CA125 levels. Otherwise, we could monitor the recurrence though the combination of HE4 and CA125 during the follow-up. And in multivariate analysis, patients with higher serum HE4 levels during follow-up had 15.565 times risk for recurrence, 4.493 times risk for death as much as patients with lower serum HE4 levels. Patients with higher CA125 levels during follow-up had 7.864 times risk for recurrence, 8.835 times risk for death as much as patients with lower serum HE4 levels.

HE4 is related to the occurrence and development of EC. The prognosis of patients with EC is poor with the gene mutation. The expression levels of HE4 in EC tissue is significantly higher than those in normal endometrium or benign endometrial lesions. The same is true for serum HE4 levels. Moreover, serum HE4 levels are associated with the tumor load, which is significantly higher before primary treatment than that after primary treatment, and it will elevate with the recurrence^10,15^.

Previous studies had shown that HE4 and CA125 could be used as biomarkers to distinguish patients with EC from benign lesions, with good sensitivity and specificity, especially the cut-off value for HE4 between 60 and 70 pmol/L. Yilmaz et al. found that the cut-off level for preoperative serum HE4 of 60.95 pmol/L was associated with a sensitivity and specificity of 72.7% and 84.4% for the diagnosis of EC, respectively^16^. Dewan et al. reported that a sensitivity and specificity of 86.7% and 100% for preoperative serum HE4 of 69.72 pmol/L to identify EC^17^. The HE4 and CA125 levels were related to tumor heterogeneity. In the previous studies, patients with later stage, poorer differentiation, deep myometrial invasion and lymph node metastasis had higher HE4 and CA125 levels^9,18–20^. Our results were in agreement with them.

However, there were relatively few studies on type II EC. Type II EC has a worse prognosis, and is more prone to recurrence. Early diagnosis and monitoring of recurrence, and timely intervention are essential. In the present study, we showed that the preoperative serum HE4 levels could be used to predict recurrence of patients. For the high-risk patients, postoperative follow-up should be enhanced. In addition, HE4 and CA125 levels during the follow-up could dynamically reflect the tumor load to monitor the recurrence^10^. HE4 and CA125 have been used as biomarkers for ovarian cancer for decades. Recently, many studies have proposed that HE4 and CA125 could be applied to early diagnosis and monitor the recurrence for EC. A previous study reported that in the multivariate analysis, preoperative serum HE4 levels exceed 81 pmol/L is an independent factor for recurrence^21^. In our study, a preoperative serum HE4 levels greater than 89.50 pmol/L was associated with a sensitivity and specificity of 61.1% and 72.1% for predicting recurrence, respectively. However, few studies proposed that the role of HE4 and CA125 in monitor recurrence during follow-up after treatment. A study showed that HE4 levels increased during follow-up greater than 70 pmol/L, with a sensitivity and specificity of 81% and 64% for predicting recurrence, respectively^10^. Our study showed that CA125 levels rose again exceed 32.45 U/mL may indicate recurrence, with a sensitivity and specificity of 71.0% and 93.4%, respectively. For HE4 levels, it was greater than 92.50 pmol/L, with a sensitivity and specificity of 61.3% and 96.2%, respectively.

CA125 is highly susceptible to other factors, such as menstruation, pregnancy, endometriosis and peritonitis^22^. HE4 is relatively stable, but also affected by smoking, renal failure and so on^11,23,24^. We found that the combination of the two had a better effect in monitoring recurrence, with a sensitivity and specificity as high as 80.6% and 91.5%, respectively. In addition, we also found that the cut-off value obtained from ROC curve could be used to predict DFS and OS of patients, suggesting that HE4 could be used as a biomarker to predict the prognosis of patients of EC.

As expected, this retrospective study attempts to use HE4 and CA125 for early detection of recurrence, which could precede clinical and imaging manifestation, so that we could diagnose and treat it in time.

A large-scale multi-institutional prospective future research is required to support the value of HE4 and CA125 for the monitoring recurrence of EC.

## Conclusion

The preoperative serum HE4 levels could be used to evaluate the risk factors of type II EC, such as later stage, poorer differentiation, deep myometrial invasion and lymph node metastasis, and higher HE4 levels were relative to worse DFS and OS. Which suggested pretreatment HE4 levels might associated with the prognosis of type II EC. What’s more, the combination of HE4 and CA125 could be used to monitor recurrence during follow-up, which contributes to earlier detection and intervention treatment without delay.

## Data Availability

The datasets generated during and/or analysed during the current study are available from the corresponding author on reasonable request.
